# Slit-Surface Electrospinning: A Novel Process Developed for High-Throughput Fabrication of Core-Sheath Fibers

**DOI:** 10.1371/journal.pone.0125407

**Published:** 2015-05-04

**Authors:** Xuri Yan, John Marini, Robert Mulligan, Abby Deleault, Upma Sharma, Michael P. Brenner, Gregory C. Rutledge, Toby Freyman, Quynh P. Pham

**Affiliations:** 1 Arsenal Medical, Inc., Watertown, Massachusetts, United States of America; 2 School of Engineering and Applied Science, Harvard University, Cambridge, Massachusetts, United States of America; 3 Department of Chemical Engineering, Massachusetts Institute of Technology, Cambridge, Massachusetts, United States of America; Universita' degli Studi del Salento, ITALY

## Abstract

In this work, we report on the development of slit-surface electrospinning – a process that co-localizes two solutions along a slit surface to spontaneously emit multiple core-sheath cone-jets at rates of up to 1 L/h. To the best of our knowledge, this is the first time that production of electrospun core-sheath fibers has been scaled to this magnitude. Fibers produced in this study were defect-free (i.e. non-beaded) and core-sheath geometry was visually confirmed under scanning electron microscopy. The versatility of our system was demonstrated by fabrication of (1) fibers encapsulating a drug, (2) bicomponent fibers, (3) hollow fibers, and (4) fibers from a polymer that is not normally electrospinnable. Additionally, we demonstrate control of the process by modulating parameters such as flow rate, solution viscosity, and fixture design. The technological achievements demonstrated in this work significantly advance core-sheath electrospinning towards commercial and manufacturing viability.

## Introduction

Electrospinning of micro and nanofibers has been investigated for decades, but the formation of core-sheath fibers by electrospinning is a relatively recent innovation. The core-sheath electrospinning process is unique in that it allows the fabrication of fibers that are particle-encapsulating, bicomponent, hollow, or not normally electrospinnable [1, 2]. These types of fibers have application across multiple industries. For example, many groups have demonstrated the capability to encapsulate compounds such as small molecule drugs, proteins and growth factors, nucleic acids, liposomes, etc. for controlled drug delivery and/or tissue engineering applications [3–6]. Other researchers have employed core-sheath electrospinning to create bicomponent fibers with novel self-cleaning, self-healing, or superhydrophobic properties that could revolutionize the textile and filtration industries [7–9]. Hollow fibers with high surface-to-volume ratios have been proposed for use in microfluidics, photonics, energy storage, and sensor applications [10]. Finally, many polymers are challenging to process into a fibrous form factor (e.g. low molecular weight, flowable materials) and are therefore not electrospinnable on their own; however, core-sheath electrospinning imparts the ability to process these unelectrospinnable materials into fibrous form factors for novel applications [11–13]. It is this versatility in core-sheath fiber structure and composition that has spurred the design and fabrication of novel electrospun materials, generating an abundance of excitement in this field.

However, production of core-sheath fibers by electrospinning at commercially viable throughputs has been a significant challenge. Typically, fabrication of core-sheath fibers from needle-based electrospinning systems is achieved in one of two ways: (1) spinning from emulsions or (2) use of a coaxial needle. Emulsion-based electrospinning exploits the inherent surface energies of two different polymeric solutions as a driving force for spontaneous organization into a core-sheath fiber architecture [14]. A limitation of this technique is the paucity of material systems which form emulsions, are electrospinnable, and organize into core-sheath fibers. Coaxial electrospinning is a process in which two polymer solutions are ejected in a core-sheath architecture from concentrically positioned needles toward a collector. This method is amenable towards a much broader range of materials systems. However, typical volumetric flow rates approach only in the tens of mL/h at the higher end; polymer mass rates are on the order of 0.01–0.1 g/h [15]. Various groups have addressed this limitation by developing setups that use multiple needles or that take advantage of the concept of free liquid surface electrospinning, such as the Nanospider by Elmarco [16–19]; however, these methods currently do not provide a commercially viable manufacturing option for high throughput production of electrospun core-sheath fibers. Multi-coaxial nozzle systems suffer from complexity and maintenance challenges, making this approach cost-prohibitive [20]. Recently, Forward et al. demonstrated electrospun core-sheath fiber fabrication at higher throughputs relative to a needle by rotating wires through two solutions. Specifically, wire electrodes were immersed into two immiscible liquids layered on top of each other to form core-sheath droplets that eventually electrospin into fibers. While this method shows promise, it requires the sheath and core solutions to have limited miscibility [21]. Here, we describe an approach, henceforth referred to as “slit-surface electrospinning”, which maintains both versatility across many materials systems and increases the manufacturing throughput of core-sheath micro and nanofibers by over 1,000-fold compared to traditional methods.

## Experimental

85/15L poly(lactic-co-glycolic) acid (molar percentage of 85% lactic acid and 15% glycolic acid, inherent viscosity = 1.7–2.6 dl/g in chloroform, 85/15L-PLGA) and 75/25L poly(lactic-co-glycolic) acid (molar percentage of 75% lactic acid and 25% glycolic acid, inherent viscosity = 2.63 dl/g in chloroform, 75/25L-PLGA) were purchased from PURAC Biomaterials; polycaprolactone (PCL) (M_n_ = 80,000 g/mol), poly(ethylene oxide) (PEO) (M_v_ = 400,000 g/mol), Nylon 6/6 (M_w_ = 262.35 g/mol), and 2,2,2-trifluoroethanol (TFE) were purchased from Sigma-Aldrich. Dexamethasone used for particulate encapsulation was purchased from Alfa-Aesar. 1,1,1,3,3,3-Hexafluoro-2-propanol (HFIP) was purchased from Oakwood Products, Inc., and the other organic solvents such as chloroform and methanol were purchased from Fisher Scientific. Silicone fibers were fabricated using polydimethylsiloxane (PDMS) Sylgard 184 kit. Prepolymer solutions were mixed according to manufacturer’s recommendations.

All the polymer solutions were made at room temperature. Six different materials systems were used in the experiments described in this communication and are described in Table 1. The use of dexamethasone served a dual purpose, as a demonstration of particulate encapsulation and as a tracer material to visualize the stream of core solution in the cone-jets. Hollow fibers were fabricated by immersing the electrospun fibers in distilled-deionized water to dissolve out the core PEO polymer, leaving behind a PLGA shell. A Nylon:PLGA pair was chosen as an example of a non-biodegradable:biodegradable bicomponent composite fiber. PDMS is a low molecular weight silicone-based organopolymer that is flowable at room temperature and therefore cannot be readily electrospun into fibers by itself. PDMS fibers could be fabricated by coaxially electrospinning a sheath polymer around a PDMS core. Subsequently, the PDMS core is allowed to cure (either at room temperature or accelerated under heated conditions), leading to solidification of the PDMS core. The result is a bicomponent fiber consisting of the sheath polymer (in this case, PLGA) and PDMS. The sheath can then be optionally removed to achieve pure PDMS fibers.

**Table 1:** Materials systems used for different experiments.

System	Core/sheath fiber type	Sheath solution	Core solution
A	Particulate encapsulation	3.5 wt% 85/15 L-PLGA in HFIP	12 wt% PCL:dexamethasone (70:30) in 6:1(v/v) chloroform:methanol
B	Particulate encapsulation	5.5 wt% 75/25 L-PLGAin TFE	12 wt% PCL:dexamethasone (70:30) in 6:1(v/v) chloroform:methanol
C	Particulate encapsulation	12 or 16 wt% PCL in 6:1(v/v) chloroform:methanol	12 wt% PCL:dexamethasone (70:30) in 6:1(v/v) chloroform:methanol
D	Bicomponent	7 wt% Nylon in HFIP	3 wt% 85/15 L-PLGAin HFIP
E	Hollow	3 wt% 85/15 L-PLGAin HFIP	2 wt% Poly(ethylene) oxide

The high voltage DC power supply used in these studies was an ES100 unit with maximum of 10 W output, purchased from Gamma High Voltage Research. Depending on the experiment, the applied voltage ranged from 70–90 kV. A 30 cm by 30 cm aluminum plate was placed approximately 50 cm above the slit fixture to collect electrospun fibers. The collector was grounded to form an electric field between the charged slit fixture and collector and to dissipate charges carried by the collected fibers. Viscosities of the solutions were measured using a Brookfield viscometer (model: LVDVE; spindle: #31). Digital images of core-sheath cone-jets were captured by a Basler A601f CMOS camera with a modified lens by attaching a 10 cm C-mount extension tube to a Sigma 70–300 mm zoom lens. Electrospun fiber meshes were sputter-coated with palladium/gold for 1 minute and observed with a scanning electron microscope (JSM-6390, JEOL USA) at an accelerating voltage of 5 kV to evaluate fiber morphology. Cross-sections of fibers were obtained by immersing the fiber meshes in liquid nitrogen and cutting with scissors.

## Results and Discussion

### Slit-surface electrospinning technology and capability

**Fig 1:**
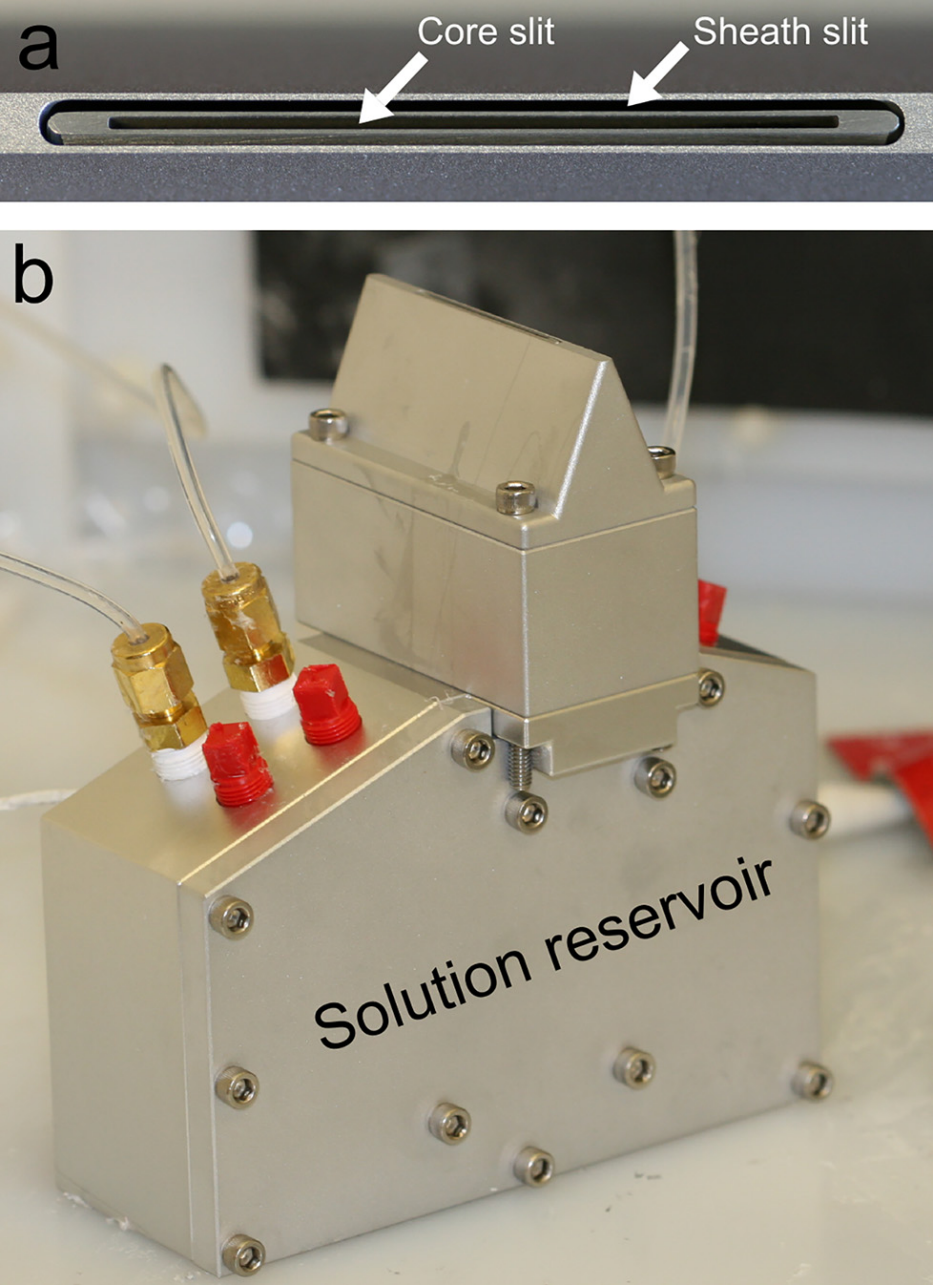
Digital images of the slit-fixture. (a) Close-up of the slit-surface created by the central alignment of two troughs. (b) Slit-fixture connected to a solution reservoir base. (c) Schematic of the axial cross section of the slit-fixture illustrating compartmentalization of sheath and core solutions below the slit and co-localization of the solutions at the slit exit.

Our electrospinning technology relies on the physical co-localization of two flowable materials along one dimension formed by a “slit-surface” resulting in multiple core-sheath jets when an electric field is applied. The slit-surface is formed via the alignment of two triangular-shaped nozzles along a single vertical plane (**Fig 1)**. Core and sheath polymer materials are delivered to the slits through their respective nozzles ensuring the co-localization of core and sheath materials (and subsequent core-sheath fiber formation) as they exit the nozzle. The application of a slit-surface has a significant impact on the ability to electrospin at higher flow rates. In our initial 3.5 cm long slit-surface design, approximately 8 electrospinning jets would form to accommodate a maximum flow rate of 0.25 L/h (at higher flow rates, quality fibers could not be obtained). Subsequently, we have scaled the length of the slit-surface to 14 cm, which resulted in the formation of 30 or more jets, enabling us to operate at total flow rates in excess of 1 L/h while producing quality fibers. At typical solution concentrations ranging from 3 to 20 wt%, these volumetric flow rates are equivalent to polymer mass rates of 50–250 g/h. To the best of our knowledge, this represents the first time that core-sheath micro and nanofibers have been fabricated via electrospinning at throughputs exceeding 1 L/h.

**Fig 2:**
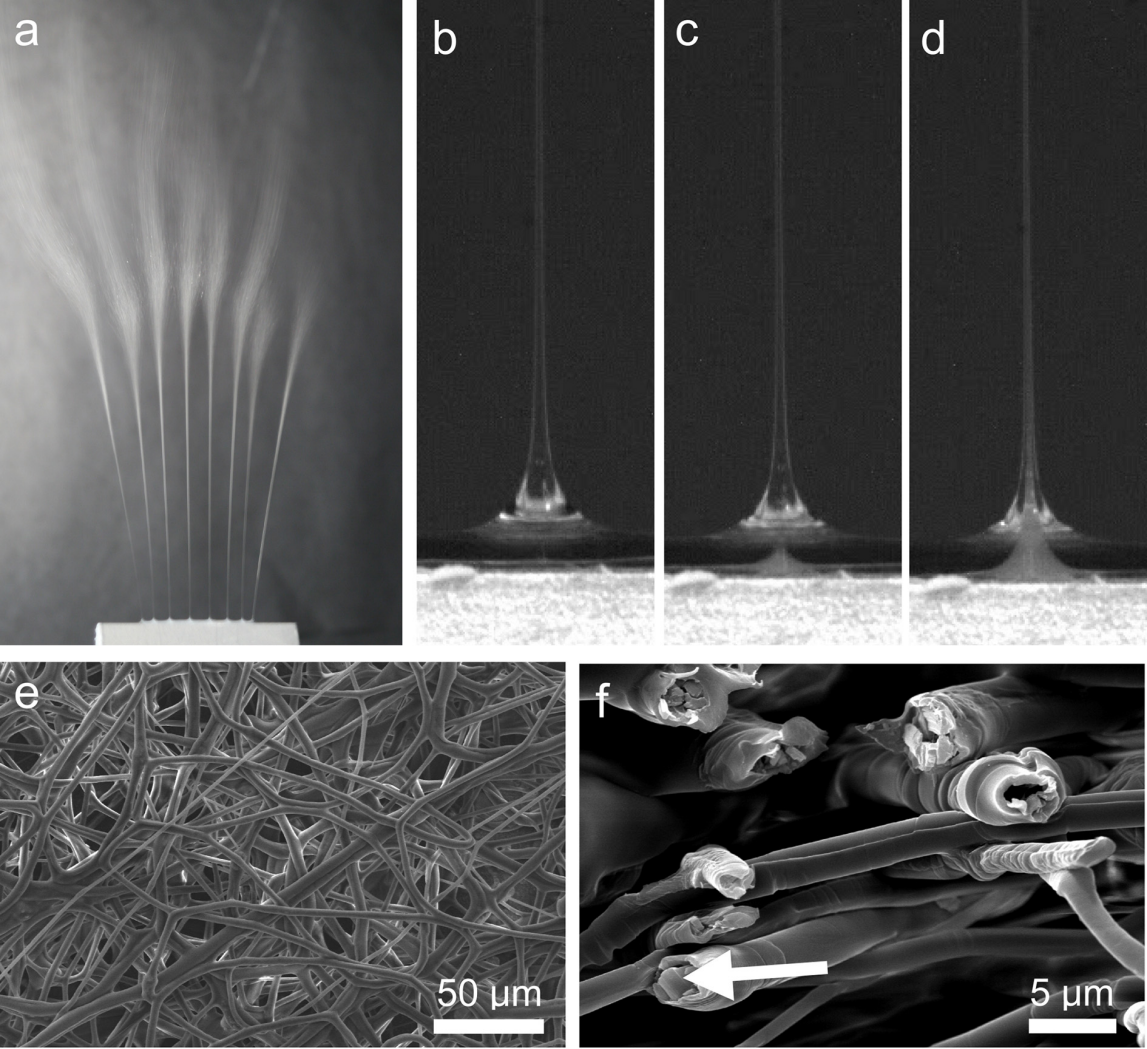
Overview of slit-surface electrospinning using System A. (a) Example of multiple electrospinning cone-jets formed across a slit-surface. (b) Electrospinning jet formed from sheath solution without core solution entrainment. (c) Same electrospinning jet as in (b), demonstrating the spontaneous entrainment of core solution. (d) Fully-formed electrospinning jet exhibiting a core-sheath structure. (e) Representative scanning electron microscopy image of fibers fabricated using slit-surface electrospinning. (f) Cross-sectional image showing core-sheath fiber structure. The arrow points to a dexamethasone drug particle.

The fundamental operation of slit-fixture electrospinning is similar to needle-based systems. The fixture itself is connected to a high-voltage source for generation of an electric field. Upon application of a critical electric field strength, multiple Taylor-like cone-jets initiate along the length of the slit-surface as shown in Fig 2A. As the core and sheath materials exit from their respective slits, they spontaneously form multiple core-sheath cone-jets that ultimately lead to core-sheath fibers (Fig 2B–2D). Initially, the cone-jet is composed of only the sheath material, but within a few milliseconds to seconds, a core-sheath cone-jet forms (Fig 2B). We hypothesize that the internal fluid pressure drops at the locations where sheath jets are present. As a result, the inner core solution (rendered white in Fig 2 due to the presence of a dexamethasone particulate tracer) preferentially flows towards locations with lower relative pressure. This observation is similar to a well-known fluid dynamics phenomenon termed “selective withdrawal”, a process referring to flow at or near stratified layers of fluids induced by suction through a tube that is immersed in the upper fluid layer. At low flow rates, there is insufficient fluid shear stress, resulting in only the upper fluid being withdrawn; however, at higher flow rates, there is enough shear stress generated such that a spout forms at the interface, and both fluids are withdrawn into the tube [22–24]. In our process, we hypothesize that viscous shear forces generated from flow of the electrospun sheath solution entrain the core solution, at the interface, to form a core-sheath cone-jet. The viscous shear force can be manipulated by adjusting variables such as solution flow rate, solution viscosity, and nozzle geometry to control the core-sheath structure. We have observed that this phenomenon occurs independently of the miscibility between the core and sheath solutions within the set of process parameters tested (see System C). We believe this to be true when the shear forces are sufficient for core material entrainment and the contact time between the two solutions at the interface is sufficiently short so as to not allow mixing between the two solutions. Fig 2E and 2F show representative images of a typical fiber mesh and evidence of core-sheath fibers encapsulating dexamethasone, respectively.

**Fig 3:**
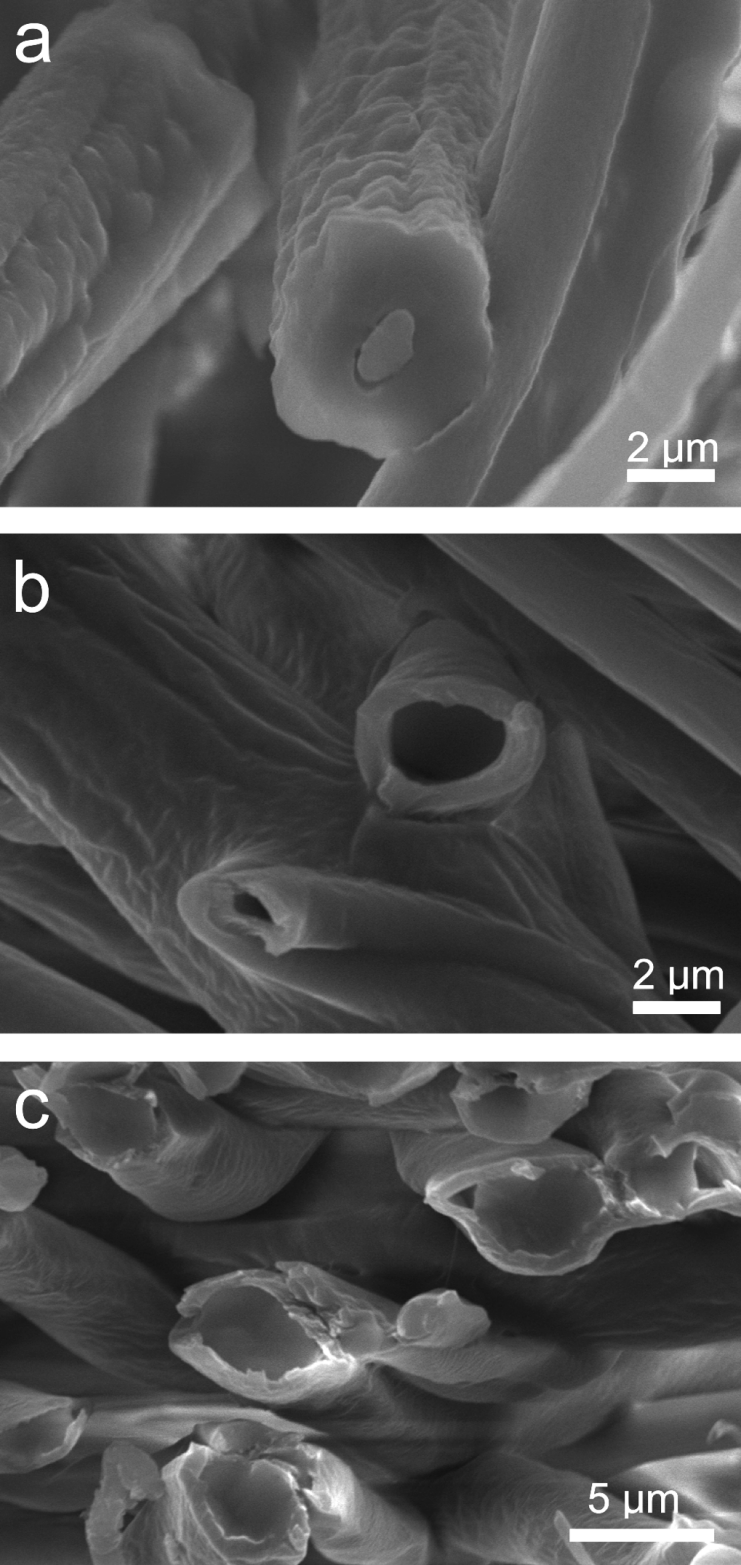
Representative scanning electron images depicting different types of core-sheath fibers fabricated using slit-surface electrospinning. (a) bicomponent (System D) (b) hollow (System E); and (c) unelectrospinnable PDMS core—PLGA sheath (System F).

Physical co-localization of core and sheath solutions by the slit surface increases versatility across materials systems. As a demonstration of this capability, we have also fabricated bicomponent, hollow, and polydimethylsiloxane (not normally electrospinnable) fibers from various different materials systems (see Experimental section for specific details of material systems used). As shown in Fig 3, cross-sections of these fibers distinctively and clearly depict a core-sheath architecture for each fiber type produced. This result is significant, suggesting that a multitude of polymers can be successfully electrospun into core-sheath fibers using our novel slit-surface fixtures (much like coaxial needle electrospinning). Indeed, the current library of materials that have been successfully electrospun so far using our technology include core/sheath polycaprolactone /poly(lactic-co-glycolic) acid, poly(ethylene oxide)/poly(lactic-co-glycolic) acid, poly(lactic-co-glycolic) acid /nylon, polydimethylsiloxane /polyvinylpyrollidone, hydroxypropyl methylcellulose /polyvinlypyrollidone, polyvinylpyrollidone /cellulose acetate, and various polyurethanes. This capability allows for flexibility in material system selection, enabling impact across multiple industries (e.g. textile, medical, energy, diagnostics, etc.).

### Control of core-sheath cone-jets

We believe that a requisite for a core-sheath fiber architecture is the formation of a core-sheath cone-jet where conditions maintain a clear distinction between the core and sheath electrospun fluids. From this perspective, we performed a series of experiments to identify and understand the variables and conditions under which distinct core-sheath cone-jets form using our novel fixture design. We have determined that (1) flow velocities at the slit exit and (2) viscosities of the solutions are major parameters in the formation of distinct core-sheath cone-jets. Solution miscibility was not a major factor. In fact, we have successfully formed core-sheath fibers from the same polymer and solvent systems using this process. For these experiments, we used one of three materials systems described in Table 1 (System A, B, or C) to visualize the morphology of the core-sheath cone-jets. (Note: Initial design of the slit-surface focused on demonstrating proof-of concept. Therefore, the experiments described herein were conducted using our initial prototype 3.5 cm design prior to scaling to a 14 cm design that allowed for a 1 L/h throughput benchmark).

#### Impact of solution flow velocity on formation of core-sheath cone-jets

**Fig 4:**
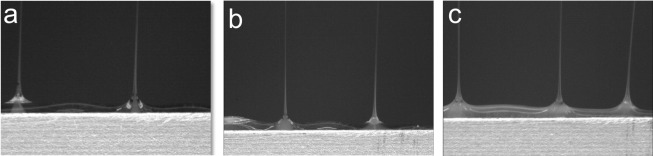
Video capture images depicting the morphology of core/sheath cone-jets using System A. Experiments were conducted at a constant sheath flow rate of 200 ml/h while varying the core solution flow rate. (a, b) Distinct core/sheath cone-jets were formed when the core flow rates were set to 40 and 20 ml/h, respectively. (c) Non-distinct core/sheath cone-jets were formed when the core flow rate was set to 60 ml/h.

**Fig 5:**
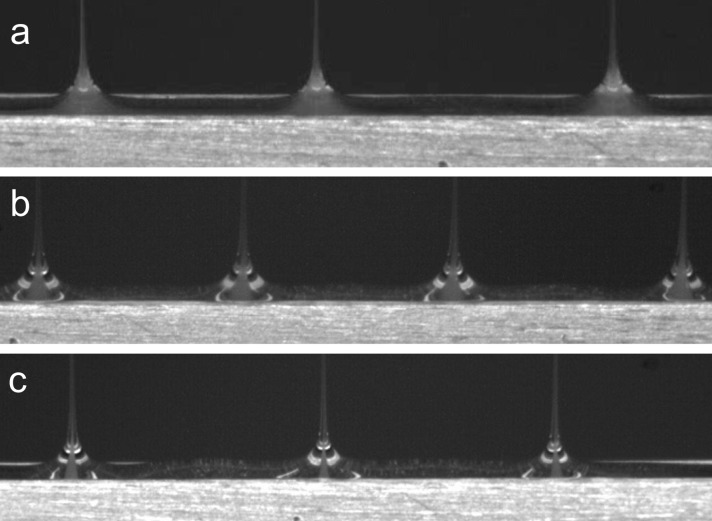
Video capture images depicting the morphology of core/sheath cone-jets of System B. Experiments were conducted under different solution flow rates to demonstrate control of emitted core-sheath cone-jets. The core fluid stream narrows as the ratio of total solution velocity to core solution velocity is increased. (a) v_sheath_:v_core_ = 2.0, (b) v_sheath_:v_core_ = 5.5, and (c) v_sheath_:v_core_ = 7.0. (Width of image corresponds to field of view of 17 mm).

Solution flow velocity at the slit exit is dependent on and can be manipulated by changing the solution flow rate and/or surface area of the core and sheath slits. As a first order estimation, we defined sheath flow velocity at sheath slit exit, v_sheath_ = (sheath flow rate+core flow rate)/surface area of sheath slit and core flow velocity at core slit exit, v_core_ = core flow rate/surface area of core slit. Using System A, the effect of solution flow rate on core-sheath cone-jet formation was investigated by keeping the sheath solution flow rate constant at 200 mL/h while varying the core solution flow rate to 20, 40, and 60 mL/h. As shown in Fig 4A–4C, distinct core-sheath cone-jets were observed only when the core flow rate was set to 20 or 40 mL/h (Fig 4A and 4B). The conditions with distinct core-sheath cone-jet formation corresponded to when the sheath solution flow velocity (v_sheath_) exiting the top of the sheath slit was greater than the core solution flow velocity (v_core_) exiting the top of the core slit. Similar results were obtained when the sheath solution flow rate was varied to 200, 100, or 40 mL/h while the core solution flow rate was kept constant at 20 mL/h. Again, distinct core-sheath cone-jets successfully formed only when v_sheath_ was greater than v_core_. Using these results as a guideline for electrospinning of System B, we manipulated the ratio of v_sheath_ to v_core_ to be 2.0, 5.5, or 7.2 (corresponding to sheath and core flow rates of 100/20, 300/20, and 200/10, respectively, all quantities being expressed in mL/h) in order to manipulate the width of the core solution stream in the core-sheath cone-jets. As the ratio of v_sheath_ to v_core_ increased in value, it appeared that the width of the core stream decreased in size (Fig 5). We hypothesize that this results in better encapsulation of the core materials. These results support our hypothesis that the mechanism for multiple, stable core-sheath cone-jets emitting from a slit-surface is “selective withdrawal”, as described earlier. Increasing the ratio of v_sheath_ to v_core_ via flow rate manipulation increases the fluid shear force of the sheath solution acting to entrain the core solution.

#### Impact of slit width on formation of core-sheath cone-jets

**Fig 6:**
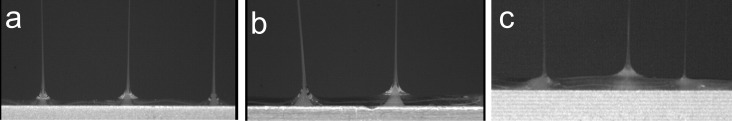
Video capture images core/sheath cone-jets using System A emitted from differing sheath slit widths. (a, b) Distinct core/sheath cone-jets were formed at slit widths of 1.5 and 2.2 mm, respectively. (c) Non-distinct core/sheath cone-jets were formed at a slit width of 3.0 mm.

Solution flow velocity is dependent not only on flow rate, but also on the cross-sectional area through which the solution flows. To demonstrate this, we investigated the impact of the sheath slit width on core-sheath cone-jet formation. The initial sheath slit width was design to be 2.2 mm. For this experiment, we examined additional sheath slits with widths of 1.5 and 3.0 mm. Sheath and core flow rates of System A were set to 200 and 20 mL/h, respectively. At these flow rates and dimensions, the ratio of v_sheath_ to v_core_ for the 1.5, 2.2, and 3.0 mm slits were 3.8, 2.6, and 1.9, respectively. For the 3.0 mm wide slit, distinct core-sheath cone-jets were not observed, while the two smaller slit-width designs resulted in distinct core-sheath cone-jets (Fig 6). Interestingly, we were able to achieve distinct core-sheath cone-jets from the 3 mm sheath slit by reducing the core flow rate from 20 to 10 mL/h (a corresponding change of v_sheath_ v_core_ from 1.9 to 3.6). These data further establish that the formation of distinct core-sheath cone-jets is facilitated by a large difference between v_sheath_ and v_core_. Similar as before, this increases the fluid shear force of the sheath fluid layer acting on the core solution at the sheath-core interface which aids in the viscous entrainment of the core solution to form a core-sheath cone-jet.

#### Impact of solution viscosity on formation of core-sheath cone-jets

**Fig 7:**
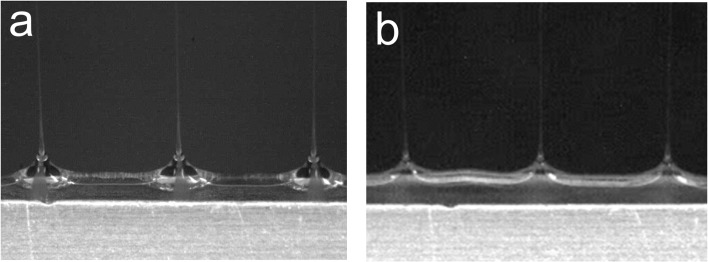
Video capture images of emitted core/sheath cone-jets using System C, whereby different sheath solution viscosities were employed. (a) Distinct core/sheath cone-jets were formed when the sheath solution viscosity was greater than the core solution viscosity. (b) Non-distinct core/sheath cone-jets were formed when the sheath solution viscosity was less than the core solution viscosity.

Solution viscosity also had a major impact on the formation of distinct core-sheath cone-jets. In this experiment, we used System C in which the sheath solution viscosity was either 280 cP or 760 cP, corresponding to PCL solutions with concentrations of 12 wt% or 16 wt%. The viscosity of the core solution was constant at 500 cP. In this experiment, the 2.2 mm wide sheath slit was used for both sheath solutions, and the flow rates were set at 200 and 20 mL/h for the sheath and core solutions, respectively. It was found that the core-sheath formation and morphology of the cone-jets was more distinct when 16 wt% PCL was used as the sheath solution, even though the same flow rates were used (Fig 7). We hypothesize that this results from the higher sheath viscosity, which provides a shear force sufficient to entrain the core solution. In contrast, the 12 wt% PCL solution has a viscosity lower than that of the core solution (280 cP < 500 cP) and does not exhibit distinct core-sheath cone-jet formation. (Note: Both conditions shown here met the conditions of sheath flow velocity being greater than core flow velocity as described in the previous section). Again, as before, we believe that this result highlights the importance of the selective withdrawal mechanism in which a sufficient shear stress is required for proper entrainment of the core material for distinct core-sheath cone-jet formation.

The data to date suggest that multiple, inter-dependent variables (solution flow rates, viscosity, and fixture design) exist that can be leveraged to control core-sheath cone-jets emitted from a slit-surface, not unlike coaxial needle electrospinning. The design of slit-surfaces and controlling operating parameters such that the shear stress between the sheath and core solutions is maximized (where the shear stress pulls the core solution into the jet) are important for formation of core-sheath jets from a slit fixture.

## Conclusion

In summary, we have developed an electrospinning process that uses novel slit-fixtures to produce core-sheath fibers at throughputs orders of magnitude higher than traditional needle-based systems. Additional benefits of the slit-fixture are less maintenance and fewer clogging issues, allowing for a more stable process when compared to an array of coaxial needles. With respect to versatility, our process is similar to needle-based coaxial electrospinning in which fibers that are particle-encapsulating, bicomponent, hollow, and unelectrospinnable could be fabricated. Control over core-sheath cone-jet morphology could be manipulated via slit size, flow rate, and solution viscosity. Versatility and process control are both attributes that are highly attractive and desirable for manufacturing operations. In on-going work, we are scaling the slit-fixtures to longer lengths, targeting even higher throughput benchmarks, and developing a system with longer or continuous run-time in order to maximize efficiency and manufacturability. In doing so, we believe that slit-surface electrospinning will fulfill the tremendous potential of electrospun core-sheath fibers by enabling their successful manufacturing and commercialization.
